# Supplementary material for: Nurse resilience, burnout, pandemic stress, and post-traumatic stress: A secondary analysis of a longitudinal cohort
Source: PLoS One. 2025 Aug 26;20(8):e0328976. doi: 10.1371/journal.pone.0328976 (PMC12380280; doi:10.1371/journal.pone.0328976)
Supplement: S1 Table — (DOCX) [file pone.0328976.s001.docx]

**S1 Table**. **Mauchly’s Sphericity Assumption Results for Aim 1.**

| **Measure** | ***X*^2^** | **df** | **Significance (*p*)** |
| --- | --- | --- | --- |
| Burnout | 3.52 | 2 | .172 |
| Resilience | .05 | 2 | .976 |
| PTS | 3.30 | 2 | .193 |
