# Supplementary material for: Nurse resilience, burnout, pandemic stress, and post-traumatic stress: A secondary analysis of a longitudinal cohort
Source: PLoS One. 2025 Aug 26;20(8):e0328976. doi: 10.1371/journal.pone.0328976 (PMC12380280; doi:10.1371/journal.pone.0328976)
Supplement: S2 Table — (DOCX) [file pone.0328976.s002.docx]

**S2 Table**. **Comparison of Participant Responses that Enrolled Early vs. Late at T1 to Work Adequacy Items, Stress Specific to COVID-19, PTS, Burnout, and Resilience.**

| **Measure** | **Early Participant Responses (May 2020- November 2020)** | **Late Participant Responses (December 2020- May 2021)** | ***p*- value** |
| --- | --- | --- | --- |
| Adequate Staffing, mean (SD) | n= 554  4.7 (2.0) | n= 210  3.4 (1.9) | <.001 |
| Adequate PPE, mean (SD) | n= 554  4.3 (1.9) | n= 210  5.0 (1.8) | <.001 |
| Adequate equipment, mean (SD) | n= 555  5.4 (1.4) | n= 210  5.2 (1.5) | .025 |
| Adequate preparations, mean (SD) | n= 555  5.2 (1.6) | n= 210  4.7 (1.8) | .002 |
| Stress Specific to COVID-19, mean (SD) | n= 542  30.7 (5.7) | n= 205  30.0 (6.3) | .140 |
| PTS, mean (SD) | n= 556  28.9 (17.3) | n= 210  28.5 (17.7) | .787 |
| Burnout at T2, mean (SD) | n= 191  42.4 (5.4) | n= 29  42.1 (6.4) | .741 |
| Resilience at T2, mean (SD) | n= 197  3.4 (.7) | n= 29  3.4 (.7) | .996 |
