# Supplementary material for: Nurse resilience, burnout, pandemic stress, and post-traumatic stress: A secondary analysis of a longitudinal cohort
Source: PLoS One. 2025 Aug 26;20(8):e0328976. doi: 10.1371/journal.pone.0328976 (PMC12380280; doi:10.1371/journal.pone.0328976)
Supplement: S3 Table — (DOCX) [file pone.0328976.s003.docx]

**S3 Table**. **Description of Participant Characteristics Who Remained in the CHAMPS Study Over Time Using Chi Square Analysis.** NR= Not Reported due to n <10.

| **Characteristic** | **T1-T2 participants**  **(n= 133)** | **T1-T4 participants**  **(n= 32)** | ***p*-value** |
| --- | --- | --- | --- |
| Age, mean (SD) | 35.9 (11.8) | 36.3 (11.7) | .850 |
| Years of work experience, mean (SD) | 10.1 (10.4) | 11.9 (10.8) | .364 |
| Gender | | | .727 |
| Female, n (%) | 127 (95) | 31 (97) |  |
| Male, n (%) | NR | NR |  |
| Employment Status | | | .219 |
| Full-Time, n (%) | 105 (79) | 22 (69) |  |
| Part-Time, n (%) | 28 (21) | NR |  |
| Relationship Status | | | .094 |
| Single, n (%) | 65 (49) | 10 (31) |  |
| Married, n (%) | 59 (44) | 21 (65) |  |
| Separated/Divorced/Widowed, n (%) | NR | NR |  |
| Highest Level of Education | | | .486 |
| Associates, Diploma, or Technical Degree, n (%) | NR | NR |  |
| Bachelor’s Degree, n (%) | 110 (83) | 24 (75) |  |
| Master’s/Doctoral Degree, n (%) | 22 (16.5) | NR |  |
| Hospital Setting | | | .731 |
| Metropolitan Hospital, n (%) | 73 (55) | 20 (63) |  |
| Regional Hospital, n (%) | 51 (38) | 10 (31) |  |
| Community Hospital, n (%) | NR | NR |  |
| Unit Setting | | | .005 |
| Emergency, n (%) | 19 (17) | NR |  |
| Intensive Care Unit, n (%) | 38 (35) | 19 (68) |  |
| Medical-Surgical Unit, n (%) | 13 (12) | NR |  |
| Inpatient COVID-19 Unit, n (%) | 40 (36) | NR |  |
| Prior Trauma: Yes, n (%) | 57 (43) | 19 (59) | .092 |
| Stress Specific to COVID-19, Mean | 31.0 | 30.7 | .813 |
| Burnout, Mean | 42.5 | 42.3 | .916 |
| Resilience, Mean | 3.4 | 3.4 | .866 |
| PTS, Mean | 32.8 | 28.5 | .172 |
| Adequate Staff, Mean | 4.7 | 5.4 | .036 |
| Adequate PPE, Mean | 4.3 | 4.1 | .575 |
| Adequate Equipment, Mean | 5.5 | 5.4 | .762 |
| Adequate Preparations, Mean | 5.2 | 5.2 | .886 |
